# Pathological, Immunohistochemical, and Bacteriological Characterization of Salmonellosis in Bearded Dragons (*Pogona vitticeps*)

**DOI:** 10.3390/pathogens15050457

**Published:** 2026-04-22

**Authors:** Matías A. Dorsch, Nicholas Crossland, Fabio Del Piero, Javier G. Nevarez, Thomas N. Tully, Maria S. Mitchell, Mariano Carossino

**Affiliations:** 1 Louisiana Animal Disease Diagnostic Laboratory, Baton Rouge, LA 70803, USAfdelpiero@lsu.edu (F.D.P.); msmitch@lsu.edu (M.S.M.); 2 Department of Pathobiological Sciences, School of Veterinary Medicine, Louisiana State University, Baton Rouge, LA 70803, USA; 3 National Emerging Infectious Diseases Laboratories, Boston University, Boston, MA 02118, USA; ncrossla@bu.edu; 4 Department of Virology, Immunology, and Microbiology, Boston University Chobanian and Avedisian School of Medicine, Boston, MA 02118, USA; 5 Department of Pathology and Laboratory Medicine, Boston University Chobanian and Avedisian School of Medicine, Boston, MA 02118, USA; 6 Department of Veterinary Clinical Sciences, School of Veterinary Medicine, Baton Rouge, LA 70803, USA; jnevare@lsu.edu (J.G.N.); ttully1@lsu.edu (T.N.T.)

**Keywords:** *Salmonella* spp., bearded dragon, sepsis, zoonosis, subspecies *houtenae*, subspecies *diarizonae*

## Abstract

*Salmonella* spp. is a major zoonotic pathogen. Although reptiles are mostly considered subclinical carriers, clinical disease may develop following immunosuppression. Clinical salmonellosis in reptiles has been extensively reported; however, the condition has been rarely described in bearded dragons (*Pogona vitticeps*). We retrospectively analyzed six cases of salmonellosis in bearded dragons and characterized the pathological, immunohistochemical, and bacteriological findings. Clinical signs and gross findings were mostly non-specific. Histological findings mainly consisted of fibrinonecrotizing enterocolitis (83.3%); necrotizing or granulomatous hepatitis (66.7%); pneumonia including bronchopneumonia or interstitial pneumonia in one case each (33.3%); tubulointerstitial nephritis with tubular necrosis (16.7%); and coelomitis (16.7%). *Salmonella enterica* subsp. *houtenae* was cultured in three cases (33.3%), whereas *S. enterica* subsp. *enterica* serovar Rissen, *S. enterica* subsp. *enterica* serovar Cotham, and *S. enterica* subsp. *diarizonae* were cultured in one case each. Intralesional bacteria were detected via immunohistochemistry in kidneys and colon in two cases (33.3%). The predominance of lesions in the intestines and liver likely reflects initial intestinal colonization followed by hematogenous dissemination to the liver. Hepatic lesions are thought to represent different stages along a continuum, progressing from acute necrosis to discrete granuloma formation. Renal and respiratory involvement was infrequent, as reported in other reptile species. Some of the isolated *Salmonella* subspecies (*S. diarizonae* and *S. houtenae*) are well-recognized causes of clinical disease in other reptile species but not previously identified in bearded dragons. This study provides a comprehensive pathological, immunohistochemical, and bacteriological characterization of salmonellosis in bearded dragons, thus raising awareness and assisting in the identification of this condition.

## 1. Introduction

*Salmonella* spp. are Gram-negative, motile, facultative anaerobic bacilli that belong to the Enterobacteriaceae family [1]. Taxonomic classification is complex with 2,600 different serotypes including both host restricted and non-host restricted [1,2]. *Salmonella* spp. are ubiquitous bacteria that are frequently found in the intestinal tract of a vast array of veterinary species and are also a major cause of disease in humans [2].

Bearded dragons (*Pogona vitticeps*) have gained increasing popularity as pets in the last decades, mainly due to their docile personality, low space requirement, and overall low maintenance [3]. As with many other reptiles, infectious, non-infectious, and degenerative conditions are common health issues in bearded dragons [4]. Several bacterial diseases including salmonellosis, mycobacteriosis, chlamydiosis, and aeromoniasis have paramount importance mainly due to their zoonotic potential [5]. Among these, reptile and amphibian-associated salmonellosis has emerged as a major public health issue with approximately 74,000 cases reported each year in the USA alone [6].

Even though reptiles are subclinical carriers of *Salmonella* spp., clinical disease has been reported sporadically, often as opportunistic disease associated with other comorbidities or stressful factors [7,8]. However, given the fragmented information available on this condition, its epidemiology and clinical relevance are mostly unknown. Likewise, pathological characterization in bearded dragons is largely lacking, with only one report describing lesions induced by *Salmonella enterica* subsp. *enterica* serovar Brandenburg in three individuals [4]. Thus, the aim of this work is to describe the pathological, immunohistochemical, and bacteriological findings of naturally occurring salmonellosis in six bearded dragons.

## 2. Materials and Methods

### 2.1. Case Selection

We carried out a retrospective search using the database of the Louisiana Animal Disease Diagnostic Laboratory (LADDL), Louisiana State University School of Veterinary Medicine, encompassing the period 2012–2025. The search criterion included autopsies in bearded dragons with positive *Salmonella* spp. cultures. For each case, we collected information about age, sex, clinical signs, duration of illness, and whether the animal died naturally or was euthanized for reasons related to veterinary care. After the initial selection process, we reassessed the gross, histological, and bacteriological findings of each case to rule out cases with potential subclinical infections.

### 2.2. Gross and Microscopic Examination

Routine postmortem examination had been conducted in all cases with tissue samples fixed in 10% neutral buffered formalin for 24–48 h, embedded in paraffin, and cut into 4–5 μm sections. All sections were stained with hematoxylin and eosin following a standard laboratory procedure and reviewed by two or more of the authors (MAD, NC, FDP, MC). The histologic lesions for each tissue were graded following a standard score system that includes four categories based on severity: mild, moderate, marked, and severe (Supplementary Table S1).

### 2.3. Bacteriology and Serotyping

Fresh samples of one or multiple tissues per case including liver (4), lungs (2), kidney (1), coelomic swab (1), and feces (1) were submitted for aerobic and/or *Salmonella* culture (Supplementary Table S1). In all cases, samples were directly inoculated onto tryptic soy agar (TSA) with 5% sheep blood agar (Remel, Lenexa, KS, USA) and MacConkey agar (Remel, Lenexa, KS, USA). Blood agar plates were incubated aerobically at 37 °C with 5% CO_2_ for 18–24 h, while MacConkey agar plates were incubated aerobically at 37 °C for 18–24 h.

In all cases except for case 5, samples were inoculated into Tetrathionate Broth with Brilliant Green (Hardy Diagnostics, Santa Maria, CA, USA) for selective enrichment. Immediately prior to inoculation, 0.2 mL of iodine solution was added to each broth tube according to the manufacturer’s instructions. After overnight aerobic incubation at 37 °C, enrichment broths were vortexed and subcultured onto xylose lysine tergitol 4 (XLT-4) agar (Hardy Diagnostics, Santa Maria, CA, USA), followed by aerobic incubation at 37 °C for 18–24 h. Depending on the case, samples underwent direct plating, selective enrichment, or both culture methods.

Bacterial colonies were subsequently screened using a Matrix-Assisted Laser Desorption/Ionization (MALDI) Biotyper Compass (Bruker Daltonics, Bremen, Germany) for identification using the direct transfer method. Identifications were made using Bruker’s database. Identification scores range from 0.0 to 3.0, and a score ≥ 2.0 was used for species-level determination. All the *Salmonella* spp. isolates were submitted to the National Veterinary Service Laboratories of the USDA for serotyping.

### 2.4. Immunohistochemistry

We performed immunohistochemistry in selected sections from each case, including lungs, liver, kidneys, stomach, small and large intestines, cloaca, esophagus, and trachea (Supplementary Table S1). Immunohistochemistry was performed using the Polymer Refine Red Detection kit in the Leica BOND RXm platform (Leica Biosystems, Deer Park, IL, USA). Following automated deparaffinization of 4 µm tissue sections on positively charged slides (SuperFrost, VWR, Radnor, PA, USA), heat-induced epitope retrieval was performed using a ready-to-use EDTA-based buffer (pH 9.0, Leica Biosystems) at 100 °C for 20 min. Subsequently, tissue sections were incubated with an anti-*Salmonella* lipopolysaccharide (LPS) core-specific mouse monoclonal antibody (ViroStat, Cat# V6371) diluted at 1:50 in antibody diluent (Leica Biosystems) for 30 min at room temperature. Tissue sections were incubated with a polymer-labeled goat anti-mouse IgG conjugated to alkaline phosphatase for 30 min at room temperature. Finally, Fast Red was applied for 10 min at room temperature followed by a hematoxylin counterstain for 5 min. Tissue sections were coverslipped using Micromount (Leica Biosystems). The primary antibody used was validated using *Salmonella* sp.-infected tissue, which was used as a positive assay control, while omission of the primary antibody incubation step was used as negative assay control.

## 3. Results

### 3.1. Signalment and Clinical Signs in Selected Cases

From January 2012 to December 2025, 157 bearded dragons were submitted to LADDL for diagnostic workup. Of these, *Salmonella* spp. was cultured in 10 cases (10/157; 6.4%); however, only 6 of these cases (6/157; 3.8%) had gross or histological lesions consistent with salmonellosis (Table 1).

### 3.2. Gross and Microscopic Examination

Gross findings during autopsy were either absent or non-specific (Table 2), with fibrinonecrotizing colitis presumptively diagnosed grossly in one case.

**Figure 1:**
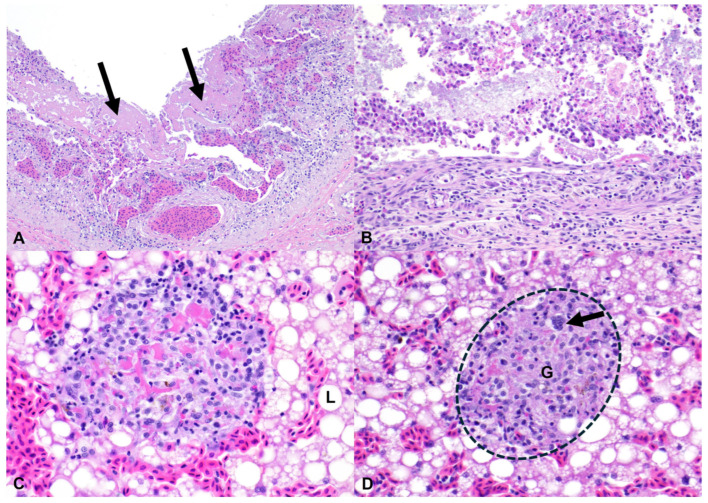
Salmonellosis in bearded dragons (*Pogona vitticeps*), H&E: (**A**) The mucosa of the small intestine is replaced by a diphtheric membrane (arrows) composed of abundant fibrin, numerous heterophils, and necrotic debris. (**B**) The colonic mucosa is ulcerated, with the underlying lamina propria infiltrated with numerous granulocytes, macrophages, and lymphocytes. (**C**) The hepatic parenchyma has a focal area of acute necrosis composed of necrotic hepatocytes, numerous heterophils, fewer histiocytes, and fibrin. Hepatocytes contain intracytoplasmic, clear, well-demarcated vacuoles that peripheralize the nucleus corresponding to lipid storage (L), a feature commonly observed in the liver of reptiles. (**D**) The hepatic parenchyma has a discrete granuloma (G; circled) composed of numerous epithelioid macrophages and a single multinucleated giant cell (arrow).

Histological findings were more frequent in the lower gastrointestinal tract (5/6; 83.3%) and were characterized by heterophilic or lymphocytic enterocolitis with infrequent intralesional short rods, sporadic ulceration, and occasional diphtheritic membranes composed of fibrin with degenerate leukocytes, necrotic debris, and extravasated erythrocytes covering the mucosa (Figure 1). The liver was frequently affected (4/6; 66.7%) with either acute or subacute to chronic histologic lesions. The acute alterations were primarily characterized by multifocal random necrotizing hepatitis with associated heterophilic and histiocytic cellular infiltrates, and occasional fibrin exudation (Figure 1). The subacute to chronic histologic lesions in the liver were characterized by granulomatous inflammation with epithelioid macrophages accompanied by few lymphocytes and scant heterophils forming distinct granulomas (Figure 1). Portal areas were infrequently expanded by low numbers of lymphocytes and histiocytes, and fewer heterophils.

**Figure 2:**
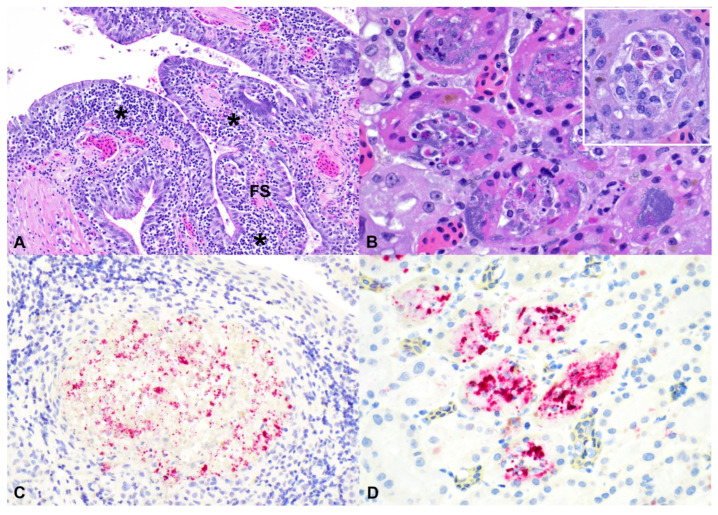
Salmonellosis in bearded dragons (*Pogona vitticeps*), H&E: (**A**) The faveolar septa (FS) are expanded by multiple small inflammatory aggregates (*) composed of lymphocytes and fewer heterophils. (**B**) The renal tubules are lined by epithelial cells exhibiting hypereosinophilia, pyknosis/karyorrhexis, and sloughing. Within the tubular lumina are numerous macrophages containing numerous intracytoplasmic short rods. Inset: details of a single tubule containing macrophages with intracytoplasmic short rods. (**C**) Bacteria are immunolabeled with an anti–*Salmonella* spp. LPS core antigen monoclonal antibody in the colon and (**D**) in the renal tubules (Fast Red).

The respiratory system was affected in two of six cases (33.3%), with lymphocytic rhinitis, tracheitis, and bronchopneumonia in case 1, and lymphocytic and heterophilic interstitial pneumonia in case 3 (Figure 2). Renal lesions were observed in case 6 only (16.7%) and were characterized by heterophilic and lymphohistiocytic tubulointerstitial nephritis with acute tubular necrosis and intratubular and intra-histiocytic short rod-shaped bacteria (Figure 2). Finally, lymphohistiocytic coelomitis was diagnosed in case 5 only (16.7%), and it was characterized by multiple aggregates of lymphocytes and histiocytes expanding the coelomic lining.

### 3.3. Bacteriology and Serotyping

*Salmonella enterica* subsp. *houtenae* was cultured from three cases (50%), whereas *S. enterica* subsp. *enterica* serovar Rissen, *S. enterica* subsp. *enterica* serovar Cotham, and *S. enterica* subsp. *diarizonae* were cultured in one case each. Interestingly, the cases from which *S. houtenae* was cultured originated from the same colony.

### 3.4. Immunohistochemistry

An anti-*Salmonella* lipopolysaccharide (LPS) core-specific mouse monoclonal antibody was used for the detection of intralesional *Salmonella* sp. Positively immunolabeled bacteria were detected in only two cases (2/6; 33.3%), in which the colon and kidneys contained numerous intralesional, frequently intracytoplasmic, short rod-shaped bacteria associated with the underlying fibrinonecrotizing colitis and heterophilic tubulointerstitial nephritis (Figure 2). These corresponded to infections with *S. enterica* subsp. *enterica* serovar Cotham and *S. enterica* subsp. *diarizonae*. No Salmonella-specific antigen was detected in the tissues examined from other cases (4/6; 67.6%).

## 4. Discussion

The present study provides a histopathological and bacteriological characterization of salmonellosis in bearded dragons, a condition infrequently reported in the literature. In this case series, salmonellosis accounted for 3.8% (6/157) of all diagnoses in bearded dragons submitted for postmortem examination at our laboratory between 2012 and 2025. These findings support the interpretation that clinical salmonellosis is an uncommon and likely opportunistic disease in bearded dragons, as has been described in other reptile species [5,7]. Failure to induce experimental disease following oral inoculation of *Salmonella* spp. in healthy bearded dragons is consistent with this observation [9].

Disruption of the equilibrium between the host immune system and the resident microflora is thought to result from predisposing factors such as overcrowding, malnutrition, stress, poor husbandry, shipping, etc. [7,10]. Identification of any of these factors can be challenging, particularly in retrospective studies such as this one; however, three of our cases were submitted from a research colony during the same year (2019), with prolonged shipment suspected as the primary stressor. Of note, no concurrent conditions were identified in our cohort. Four cases with cultured *Salmonella* spp. were excluded from this study since the underlying diseases affecting these animals significantly confounded any histologic alterations associated specifically with salmonellosis (these included mycobacteriosis [two cases]; fungal dermatitis by *Chrysosporium* spp. with concurrent intestinal nematodiasis [one case]; and egg yolk coelomitis [one case]).

Given the reduced number of cases, no analysis to determine sex or age predilection could be performed in our cohort. The age of affected animals varied widely, ranging from 4 weeks to 8 years old. Clinical signs were mostly non-specific and likely associated with sepsis; in one case, the animal was found dead without any premonitory clinical signs, as described previously in other reptiles [10,11,12].

Unlike previous reports of salmonellosis in reptiles [8,10], gross findings in this study were overall mild and non-specific. These included hepatic and pulmonary congestion in two animals (cases 2 and 3), and hepatomegaly and icterus in one animal (case 1). Case 4 had fibrinonecrotizing colitis identified on gross examination; however, this finding could not be characterized microscopically due to severe autolysis. The remaining two cases (cases 5 and 6) lacked overt gross lesions, in agreement with a previous report of salmonellosis in bearded dragons [4].

The predominance of lesions in the lower gastrointestinal tract and liver probably reflects the intestine as the primary portal of entry for *Salmonella* spp. in reptiles, as would be expected in animals containing *Salmonella* spp. as part of the normal microbiota. Therefore, following intestinal colonization and disruption of the intestinal barrier, *Salmonella* spp. can disseminate to the liver via the portal circulation [7]. The concurrent presence of intestinal and hepatic lesions in most cases in this series supports this route as the most common pathway of infection.

The presence of lymphocytic rhinitis, tracheitis, and bronchopneumonia in one case (case 1) raises the possibility of a respiratory route of infection as an alternative portal of entry. However, given that no *Salmonella*-specific antigen was detected and that hepatic lesions were also present in this animal, the respiratory lesions were more likely secondary to another, unidentified etiologic agent. On the other hand, interstitial pneumonia was observed in case 3. The pattern and distribution of the lesions resemble those described in calves infected with *Salmonella enterica* subsp. *enterica* serovar Dublin [13] and are suggestive of septicemia, a conclusion further supported by the concurrent hepatic and intestinal lesions in this case.

Enterocolitis with diphteritic membranes is characteristic of salmonellosis across multiple species, including reptiles [8,14] and mammals [15,16]. In the present study, intestinal lesions accompanied by hepatic involvement were the most consistent findings, in agreement with previous reports [8]. Regarding the hepatic lesions, the literature describes two different lesion patterns: one characterized by multifocal to coalescing areas of coagulative necrosis, and another characterized by granulomas with a core composed of caseous necrosis [8,11]. Our findings do not conform strictly to either pattern, as they comprised multiple, random, variable sized foci of necrosis with heterophils and fibrin during the acute course, and small incipient granulomas in the subacute to chronic phase. To our understanding, both lesions are part of a continuum and therefore it is likely that mature granulomas with caseous cores and peripheral fibroplasia would have formed had the animals survived longer. Finally, a distinct hepatic lesion consistent with end-stage liver was observed in case 4; this change was considered unrelated to salmonellosis and likely reflected pre-existing hepatic injury.

Lesions identified in only one animal represented in our study cohort included tubulointerstitial nephritis and coelomitis. The renal lesions included acute tubular necrosis with intratubular bacteria, which might indicate an ascending urinary infection, with concomitant intestinal, hepatic, and systemic involvement. Renal lesions induced by *Salmonella* spp. have been seldomly described in reptiles; reported cases include granulomatous nephritis induced by *Salmonella enterica* subsp. *enterica* serovar Typhimurium in Olive Ridley Turtles [17], and fibrosing interstitial nephritis caused by *Salmonella* spp. in an iguana [12]. In the case with coelomitis, it is hypothesized that it originated from the ulcerative colitis and cloacitis.

Immunohistochemistry has been considered a useful diagnostic tool, particularly when bacterial culture is unavailable [8]. The LPS core antigen, the target of the monoclonal antibody used in this study, is highly conserved across *Salmonella* subspecies and serovars [18]. In support of this, the two cases in which positively immunolabeled bacteria were recognized in this study corresponded to *S.* Cotham and *S. diarizonae*, respectively. However, there are also reported differences in recognition of *Salmonella* LPS core antigen across subspecies and serovars associated with variation of the core antigen linked to polymorphisms in the *waa* biosynthetic locus and to steric masking of core epitopes by O-antigen chains. *S. houtenae* and smooth serovars such as *S.* Rissen exhibit either altered core antigen associated with subspecies divergence or O-antigen-mediated shielding, respectively, hindering antibody binding [19,20,21,22,23]. Thus, we hypothesize that these differences have hindered our ability to detect intralesional bacteria via immunohistochemistry in four of six cases associated with *S. houtenae* and *S.* Rissen infections. It is also possible that the sensitivity was also limited by the postmortem interval or the limited number or selection of tissues examined.

Four different *Salmonella* spp. serovars were cultured in this study. Among these, *S. diarizonae* and *S. houtenae* have previously been associated with clinical salmonellosis in snakes [8,10] and other reptiles [12,24], but not bearded dragons, making this the first report of their occurrence in this species. Scant information is available about the other two serovars identified in this study. Recently, *S.* Cotham was recently implicated in a human outbreak linked to pet bearded dragons [25], whereas *S.* Rissen was isolated from children infected following contact with aquatic turtles [26].

Finally, it is important to acknowledge some limitations in this study such as the small number of cases as well as the lack of immunohistochemical identification in a proportion of the cases.

## 5. Conclusions

This study provides a comprehensive pathologic, immunohistochemical, and bacteriologic characterization of salmonellosis in bearded dragons, therefore contributing to the scant available literature on this zoonotic condition. Our findings indicate that clinical salmonellosis primarily affects the gastrointestinal tract and liver, and to a lesser extent, the respiratory tract, kidneys, and coelom. Among the isolated *Salmonella* subspecies were *S. diarizonae* and *S. houtenae*, neither of which has previously been reported in bearded dragons. This study may serve as a useful reference for practitioners and diagnosticians, who are likely to encounter bearded dragons more frequently given their increasing popularity as companion animals.

## Figures and Tables

**Table 1:** Signalment and clinical history of bearded dragons (*Pogona vitticeps*) infected by *Salmonella* spp. included in this study.

Case	Age	Sex	Clinical Signs	Euthanasia or Natural Death
1	Adult	F	NA	Natural
2	Adult	M	NA	Euthanized
3	Adult	F	Weakness, anemia	Euthanized
4	8y	F	Anorexia, lethargy, dehydration, weight loss, weakness	Euthanized
5	4w	F	Obtunded mentation, weight loss	Natural
6	3y	M	Hyporexia, lethargy	Natural

F = female; M = male; NA = not available.

**Table 2:** Histopathological, bacteriological, and immunohistochemical findings in bearded dragons (*Pogona vitticeps*) diagnosed with salmonellosis.

Case	Gross Findings	Histology	Bacterial Culture	*Salmonella* spp. IHC
1	Icterus; hepatomegaly; pericardial effusion	Heterophilic and granulomatous hepatitis; lymphocytic rhinotracheitis; lymphocytic bronchopneumonia	*S. houtenae*	−
2	Hepatic congestion	Granulomatous hepatitis; lymphocytic and heterophilic enterocolitis	*S. houtenae*	−
3	Hepatic and pulmonary congestion	Granulomatous hepatitis; lymphocytic and heterophilic interstitial pneumonia; heterophilic and lymphocytic enterocolitis	*S. houtenae*	−
4	Fibrinonecrotizing colitis	Hepatic fibrosis with ductular reaction	*S.* Rissen	−
5	NA	Lymphohistiocytic coelomitis; ulcerative and granulocytic colitis and cloacitis	*S.* Cotham	+
6	Fibrinonecrotizing enteritis	Heterophilic and lymphohistiocytic tubulointerstitial nephritis; fibrinonecrotizing enteritis; hepatocellular necrosis	*S. diarizonae*	+

NA = not available.

## Data Availability

The original contributions presented in this study are included in the article/Supplementary Material. Further inquiries can be directed to the corresponding author.

## Supplementary Materials

The following supporting information can be downloaded at: https://www.mdpi.com/article/10.3390/pathogens15050457/s1, Table S1: Summary table of tissues processed by immunohistochemistry and *Salmonella*-specific bacteriological culture, and morphological diagnoses and scoring of relevant histological alterations.
